# Cell Type-Specific Mechanisms in the Pathogenesis of Ischemic Stroke: The Role of Apoptosis Signal-Regulating Kinase 1

**DOI:** 10.1155/2018/2596043

**Published:** 2018-03-20

**Authors:** So Yeong Cheon, Eun Jung Kim, Jeong Min Kim, Bon-Nyeo Koo

**Affiliations:** ^1^Anesthesia and Pain Research Institute, Yonsei University College of Medicine, Seoul, Republic of Korea; ^2^Department of Anesthesiology and Pain Medicine, Yonsei University College of Medicine, Seoul, Republic of Korea

## Abstract

Stroke has become a more common disease worldwide. Despite great efforts to develop treatment, little is known about ischemic stroke. Cerebral ischemia activates multiple cascades of cell type-specific pathomechanisms. Ischemic brain injury consists of a complex series of cellular reactions in various cell types within the central nervous system (CNS) including platelets, endothelial cells, astrocytes, neutrophils, microglia/macrophages, and neurons. Diverse cellular changes after ischemic injury are likely to induce cell death and tissue damage in the brain. Since cells in the brain exhibit different functional roles at distinct time points after injury (acute/subacute/chronic phases), it is difficult to pinpoint genuine roles of cell types after brain injury. Many experimental studies have shown the association of apoptosis signal-regulating kinase 1 (ASK1) with cellular pathomechanisms after cerebral ischemia. Blockade of ASK1, by either pharmacological or genetic manipulation, leads to reduced ischemic brain injury and subsequent neuroprotective effects. In this review, we present the cell type-specific pathophysiology of the early phase of ischemic stroke, the role of ASK1 suggested by preclinical studies, and the potential use of ASK suppression, either by pharmacologic or genetic suppression, as a promising therapeutic option for ischemic stroke recovery.

## 1. Introduction

Ischemic stroke is a heterogeneous neurologic disorder characterized by sudden onset and multiple environmental risk factors [1, 2]. Ischemic stroke develops as a result of complex pathomechanisms induced by a critical reduction in cerebral blood flow (CBF) caused by either sudden or gradual occlusion of cerebral arteries [3, 4]. The brain requires large amounts of oxygen and glucose from the blood for energy metabolism; thus, blockage of blood circulation causes neurologic deficits [3–5]. The epicenter of a stroke, the area of the brain with crucially impaired blood flow, is referred to as the “infarct core,” and the neighboring area is referred to as the “ischemic penumbra” or salvageable area [3–5]. Ischemia-related pathologic reactions can last for days to weeks in these areas of the brain [4]. The main pathologic changes involved in ischemic stroke are energy depletion, calcium overload, excessive reactive oxygen species (ROS) generation, inflammatory signals, and ion imbalance, all of which can lead to cell death [3, 5, 6]. These changes are severely detrimental to neuronal, glial, and endothelial cell function [5] and lead to platelet activation, reactive gliosis, immune cell activation, and neuronal cell death in the ischemic brain [3, 5, 7]. Over the last decades, various stroke models have been designed in an effort to find new therapies for stroke [8]. However, therapeutic candidates from preclinical studies have failed to translate into effective therapies [8]. In this review, we will address the underlying pathophysiology of ischemic stroke briefly, focusing on cell type-specific mechanisms generated from preclinical ischemic stroke models. Additionally, we discuss apoptosis signal-regulating kinase 1 (ASK1) as a potential therapeutic target based on preclinical testing.

## 2. ASK1

**Figure 1:**
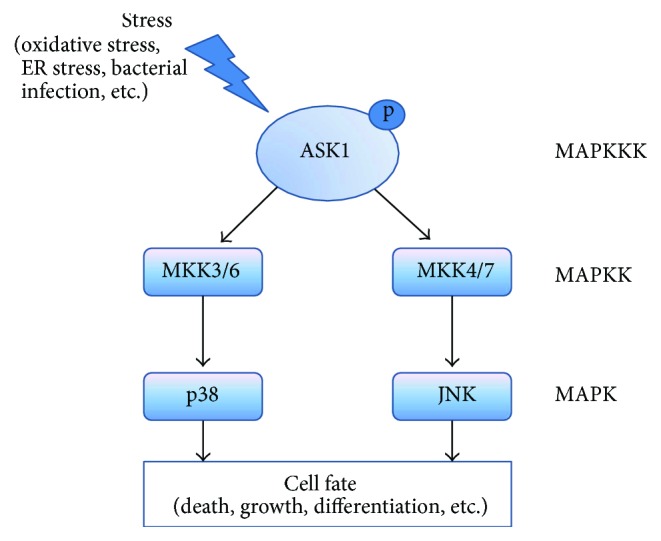
Involvement of ASK1 in the cellular mechanism. After various stresses, the activated form of ASK1 activates MAPKK (MKK3/6 and MKK4/7), thereby activating MAPK including JNK and p38. The ASK1 signaling pathway governs cellular fate such as apoptosis, growth, and differentiation.

ASK1 is a member of the mitogen-activated protein kinase kinase kinase (MAPKKK) family, which activates mitogen-activated protein kinase kinase (MAP2K: MKK4/MKK7, MKK3/MKK6) and leads to the subsequent activation of mitogen-activated protein kinase (MAPK) as part of a signaling cascade [9–12]. ASK1 is endogenously expressed in various cell types [13]. It is comprised of 1375 amino acids in humans and 1379 in mice, and it contains a serine/threonine kinase domain in the middle region [14]. The phosphorylation of threonine residues (Thr 838 in human and Thr 845 in mouse) is important for ASK1 activation [10, 15]. In normal conditions, ASK1 is a homooligomer, which binds to another ASK1 via its C-terminal coiled-coil domain. The N-terminal coiled-coil domain of ASK1 binds to thioredoxin (Trx), which suppresses ASK1 kinase activity [9, 15]. Under oxidative stress conditions, oxidized Trx is separated from ASK1, and unbound ASK1 is activated by phosphorylation [11, 16]. Calcium influx and oxidative stress can elicit phosphorylation of the ASK1 Thr residue [10, 15]. In addition, tumor necrosis factor receptor-associated factor 2 (TRAF2) and TRAF6 act as positive regulators of ASK1 after hydrogen peroxide (H_2_O_2_) injury [10]. Negative regulators of ASK1, 14-3-3 proteins, block activation of ASK1 in the steady state by binding to the C-terminal of ASK1 after Ser 966 phosphorylation [9, 10, 15]. However, oxidative stress promotes dephosphorylation of ASK1 at Ser 966 and leads to detachment of 14-3-3, which results in activation of ASK1 [10]. ASK1 is activated not only from oxidative stress but also from endoplasmic reticulum stress and bacterial infection [9, 11, 14]. Both Fas death receptor and tumor necrosis factor (TNF) also activate ASK1 [17, 18]. ASK1 is known as an early responder to ROS after cerebral ischemia [19]. After exposure to various stimuli, activated ASK1 initiates multiple signaling cascades, including c-Jun N-terminal kinase (JNK) and p38, and governs cellular mechanisms, including cell death, growth, and differentiation (Figure 1) [10, 14, 20]. Although ASK1 has previously been known to be mainly involved in apoptotic cell death [21], recent research has identified other functions of ASK1, such as its association with thrombosis, brain edema, inflammatory responses, and reactive gliosis after cerebral ischemia [19, 22–24].

## 3. Platelets and ASK1

Ischemic stroke is linked to vascular occlusion due to a thrombus or emboli in the brain [25]. Platelets are necessary for thrombosis and thromboembolism formation [26]. Platelet-induced thrombosis is associated with platelet adhesion, activation, and aggregation [27]. Normally, homeostasis is maintained by coagulation, fibrinolysis, and platelet function [28]. When homeostasis breaks down, platelets are involved in thrombus formation through a complex process [27]. For initial adhesion, platelets make connections between platelet surface receptors (glycoprotein (GP) Ib-V-IX complex or integrin *α*IIb*β*3 and *α*2*β*1) and adhesive substrates (von Willebrand factor (vWF) and collagen) on an exposed endothelial extracellular matrix (ECM) [29–31]. The platelet GP IIb/IIIa surface receptor mediates platelet aggregation through platelet-platelet interactions with extracellular fibrinogen and vWF [25, 27, 29]. These binding events trigger platelet activation and intracellular signaling pathways, which induce the production of thrombin and promote the release of activating factors (granule contents) (adenosine diphosphate (ADP), epinephrine, and thromboxane A_2_ (TXA_2_)) [27]. These factors can increase GP IIb/IIIa and calcium levels [32, 33]. Upregulated calcium can induce an increase in phospholipase A_2_(PLA_2_) [34]. A previous study proved the expression of p38, extracellular signal-regulated kinase 2 (ERK2), and JNK1 in platelets, which can be activated by thrombin, collagen, vWF, and ADP [35]. ASK1, expressed in human and murine platelets, has recently been found to be rapidly activated by different platelet agonists (ADP, convulxin, and thrombin) [22]. Genetic depletion of ASK1 leads to defects in platelet aggregation, impaired integrin *α*_IIb_*β*3 activation, and reduced TXA_2_ generation [22]. In *Ask1*^−/−^ platelets, inhibition of cytoplasmic phospholipase A2 (cPLA_2_), an important enzyme in the generation of TXA_2_, led to a reduction in thrombin-, collagen-, and convulxin-induced TXA_2_ production [22]. Impaired platelet functions, caused by ASK1 depletion in platelets, result in thrombosis deterioration, which eventually becomes protective against arterial thrombosis [22]. Hence, ASK1 serves as an important factor in thrombosis, and its activation is associated with an increased risk factor for ischemic stroke.

## 4. Endothelial Cells and ASK1

The blood-brain barrier (BBB) consists of a highly specialized endothelial structure and maintains brain homeostasis by controlling para- and transcellular transport between blood and the extracellular space [36]. Brain interendothelial junction complex contains adherence junctions (vascular endothelial cadherin, *β*-catenin), gap junctions (connexins), and tight junctions (claudins, occludins, and junctional adhesion molecules (JAM)), which are important for constructing barriers and cell-cell communication [36–39]. However, pathological conditions, such as ischemic stroke, can change BBB permeability and interactions between endothelial cells [36, 37]. A damaged BBB allows blood to enter the parenchyma and causes brain damage [40], followed by cerebral edema and vasomotor/hemodynamic dysfunctions [40].

Metalloproteinases (MMPs) and proteolytic enzymes can affect BBB integrity by degrading neurovascular substrates [37, 39]. Deterioration of the BBB caused by MMP-mediated tight junction degradation is mainly associated with MMP-9 [37]. BBB breakdown permits water into the extracellular compartments and results in vasogenic edema in the injured brain [41]. MMPs also contribute to edema formation [42]. Several reports have concluded that the excessive activation of MMP-2 and MMP-9 in the ischemic brain and cerebral endothelium leads to cellular damage [39, 43]. By inhibiting MMPs, vascular hyper permeability can be reduced by impeding degradation of gap junction proteins and rearrangement of tight junction proteins [44]. In this context, MMP-9 knockout contributes to preventing BBB disruption and enlargement of brain lesion [45].

Vascular endothelial growth factor (VEGF), a vascular permeability factor, is involved in the formation of vascular leakages and vasogenic edema [44]. VEGF has permeabilizing effects on the endothelium through MMP-9-induced reduction of tight junction proteins (zo-1, occludin) [44]. Previous studies have shown that administration of recombinant human VEGF_165_ exacerbates the breakdown of BBB integrity, which can be prevented by inhibiting VEGF at an early stage of ischemic stroke [46, 47]. A relationship between increased activation of MMP-9- and VEGF-induced BBB leakage had also been identified [48].

Although ASK1 is necessary for angiogenesis and the recovery of blood flow by direct expression of VEGF and monocyte chemoattractant protein-1 (MCP-1) after unilateral hindlimb ischemia, ASK1 and VEGF play an important role in vascular permeability in cerebral ischemia [24, 49]. Inhibition of ASK1 reduces ischemia-induced edema formation and the expression of VEGF and aquaporin-1 (water channel protein) [24]. In addition, ASK1 silencing by siRNA decreases gene levels of *Mmp3*, *Vegf-a*, *Vegf-c*, and *Aquaporin 12* and *18* [24]. ASK1 inhibition reduces MMP-9 activity in both mice and endothelial cell cultures. These results suggest a role for ASK1 in suppressing neuronal cell death [50], based on its profound effect on BBB permeability and brain edema formation after ischemic stroke.

## 5. Immune Cells and ASK1

After cerebral ischemia, circulating blood leukocytes migrate across disrupted vessel walls into the cerebral parenchyma [40]. During this influx of immune cells, adhesion molecules, such as vascular adhesion molecule-1 (VCAM-1), intercellular adhesion molecule-1 (ICAM-1), E-selectin, and P-selectin, promote the transendothelial recruitment of immune cells [51–53]. Infiltrating immune cells accumulate in the brain lesion and trigger the release of inflammatory cytokines, which further promote tissue damage [40]. Peripheral blood cells are involved in a variety of functions, from cell death to cell recovery, depending on the time course of the ischemic stroke [54].

Neutrophils are subpopulations of leukocytes, which exacerbate neuronal damage by participating in the early stages of ischemic stroke [40, 55]. Transmigrated neutrophils possess neurotoxic properties and produce cytokines, protease, chemokines, and ROS [56, 57]. Neutrophils also release neurotoxic-related neutrophil extracellular traps (NETs), composed of proteases and decondensed DNA [56]. Moreover, oxygen free radicals and proteolytic enzymes are also released from penetrated neutrophil [58]. Several studies have proven that the inhibition of either neutrophil accumulation or adhesion can diminish ischemic brain injury [55, 58]. Prevention of neutrophil infiltration toward the ischemic lesion has beneficial effects on the ischemic brain [59]. Inhibition of neutrophils by anti-neutrophil antibody (RP3) efficiently reduces the extent of brain infarction and the cerebral water content [60].

Brain microglia, representative immune cells of the brain, contribute to the immune systems in the CNS through defense mechanisms such as phagocytosis [40, 61]. After acute cerebral ischemia, microglia are activated in response to the influx of immune cells and become indistinguishable from macrophages [62–64]. Microglia are activated via the Toll-like receptor (TLR) pathway in response to cellular damage after cerebral ischemia and release cytotoxic and cytoprotective substances [40, 61]. Infiltrated blood-borne macrophages in infarcted brain tissue are key modulators of the immune system [63]. Although microglial activation leads to tissue injury during early stages of cerebral ischemia, microglia/macrophages participate in tissue recovery during the late course of ischemia [65]. Astrocytes, fibroblasts, and endothelial cells, as well as resident microglia and peripheral macrophage, are involved in the production of inflammatory cytokines such as interleukin-1*β* (IL-1*β*), transforming necrosis factor-*α* (TNF-*α*), and transforming growth factor-*β* (TGF-*β*) [58, 61, 62, 64, 66]. Although microglia and macrophages are associated with brain plasticity and recovery at later stages of cerebral ischemia, several studies have reported that microglia and macrophages induce neuronal injury through a TLR-4-dependent manner and trigger the proinflammatory mediator in the acute stages of ischemic stroke [67–69]. Pharmacological inhibition of microglia showed protective effects in cerebral ischemia by inhibiting a microglia-derived inflammatory mediator. Suppression of activated microglia by minocycline led to reduced brain infarction, improved neurological deficits, and diminished BBB leakage [70, 71]. Moreover, macrophage-derived angiopoietin-like protein 2 knockout contributed to reduced brain injury [67].

ASK1 is closely related to the immune system and is required in inflammatory responses [14, 72]. It has been reported that TLR4 activates ASK1 to initiate the MAPK pathway and thereby express inflammatory-related genes [73]. MAPK also mediates expression of a variety of inflammatory genes such as cell surface adhesion molecules, chemokines, and cytokines [74]. ASK1 is linked to ventilation-induced cytokine production, neutrophil infiltration, and cell death in the lung [75]. The TLR/ASK1/p38 pathway is important in chemokine production and in triggering neurotoxicity in multiple sclerosis [76]. ASK1 contributes to production of TNF-*α* and inducible nitric oxide synthase (iNOS) in primary microglia cell culture [77]. However, ASK1 knockout *in vivo* and *in vitro* models diminished lipopolysaccharide- (LPS-) induced upregulation of IL-6, IL-1*β*, and TNF-*α* and diminished LPS-exaggerated injury [9, 14, 72]. LPS-induced ASK1/p38 signals and cytokine production in the RAW264.7 macrophage cell line are attenuated by antioxidants [14]. From previous studies on ischemic injury, it is known that ASK1 silencing by siRNA reduces infiltrated macrophages/resident microglia in brain regions such as the striatum, cortex, and hippocampus, and ASK1 silencing downregulates proinflammatory cytokines such as IL-6, IL-1*β*, and TNF-*α* in the ipsilateral hemisphere at late stages of cerebral ischemia. In the RAW264.7 macrophage cell line and BV2 microglia cell line, ASK1 inhibition diminishes the release of proinflammatory mediators [23]. From a genetic perspective, microarray analysis shows that ASK1 silencing decreased the gene levels of *Il1b*, *Il6*, *Cxcl2*, *Cxcl1*, and *Ccl2* [19]. Considering the relationship between ASK1 and the immune response, ASK1 could be an important regulator of the inflammatory response after ischemic stroke.

## 6. Astrocytes and ASK1

The key roles of astrocytes in the neuronal system are involved in the maintenance of brain physiology and neuronal support, both structurally and metabolically, through neurotransmitter regulation (glutamate uptake/release), ion buffering, scavenging free radicals, enhancing BBB integrity, and regulating water transports [78–81]. However, astrocytes become hyperactivated in response to ischemic stress and extend their processes, changing morphology with the expression of glial fibrillary acidic protein (GFAP) [7, 78]. Astrocytes migrate toward the injury site and thus accumulate and produce a glial scar [7, 19, 80]. It has been reported that astrocytes produce and release either trophic factors (brain-derived neurotrophic factor (BDNF), fibroblast growth factor-2, and nerve growth factor (NGF)) or inflammatory cytokines (IL-6, IL-1*β*, TNF-*α*, and interferon-gamma (IFN*γ*)) [79, 82]. Trophic factors play crucial roles in neuronal survival and protection, while inflammatory mediators contribute to brain injury [18, 63, 83, 84]. Therefore, it is known that astrocytes play dual roles in the immune system [78]. Although several studies have provided evidence of the relationship between reactive astrocytes and neurogenesis, previous reports have suggested that reactive astrocytes block neuronal regeneration [19, 81, 85–87].

ASK1 is present in astrocytes and is strongly expressed after cerebral ischemia. Readily identified reactive astrocytes in ischemic lesions form a glial scar in the chronic phase of ischemic stroke, which delays extension of neurite and functional recovery [19]. However, siRNA targeting ASK1 reduced reactive astrocyte marker GFAP in both *in vivo* and *in vitro* studies, decreased glial scar formation, and promoted neuronal plasticity and functional performance [19]. Moreover, ASK1 deletion suppressed mitochondrial complex I inhibitor 1-methyl-4-phenyl-1,2,3,6-tetrahydropyridine- (MPTP-) induced astrocyte activation and protected against degeneration of dopaminergic neurons [88]. In addition, p38, a molecule downstream of ASK1, is also associated with reactive astrogliosis, and a conditional GFAP/p38 knockout reduced astrogliosis [89]. Therefore, several lines of evidence show that ASK1 may play a major role in reactive astrocytes and glial scar formation after ischemic stroke.

## 7. Neurons and ASK1

After ischemic injury, neurons are harmed by excitotoxicity, acidotoxicity, MMP, nitric oxide (NO), ion imbalances, and free radicals, which results in neuronal death and cerebral damage [90–92]. Inhibition of ATP synthesis in the mitochondria after ischemia depolarizes neuronal plasma membranes [92]. Additionally, the intracellular influx of excess calcium overloads via nonselective cation channels and calcium channels depolarizes neurons [90]. Membrane depolarization induces the release of the excitatory neurotransmitter glutamate, and the increase in glutamate concentration can activate the N-methyl-D-aspartate (NMDA) and *α*-amino3-hydroxy-5-methyl-4-isoxazolepropionic acid (AMPA) receptors [92, 93]. Calcium-permeable NMDA receptors induce further membrane depolarization, which aggravates calcium overload [92]. Ion imbalances cause excessive ROS in the intracellular system [94]. Increased NO production and free radicals can enhance BBB leakage and contribute to apoptotic signaling cascades [91].

Overexpression of ASK1 promotes apoptotic cell death, and JNK/p38 MAP kinases closely interact with this process [95, 96]. Increased ASK1 levels after cerebral ischemia also induce apoptosis, which leads to neuronal cell death and the development of infarct lesions [21]. Calcium influx activates p38 signals in the ASK1*^+/+^* mice-derived neuron, but these p38 activations are suppressed in the ASK1*^−/−^* mice-derived neuron [10]. ASK1 is closely related to Ca^2+^/calmodulin-dependent protein kinase II (CaMK II), which is activated by calcium influx [10, 97]. It has been reported that CaMKII directly phosphorylates ASK1 at Thr 838 [10, 15, 97]. CaMKII inhibition reduces Ca^2+^-induced activation of ASK1 [15, 97]. NO activates ASK1, and the nitric oxide synthase (nNOS) inhibitor 7-NI and the NMDA receptor antagonist MK801 reduce ASK1 activity [98]. In addition, an AMPA receptor blocker and a free radical scavenger prevent activation of ASK1 and JNK [99]. These previous studies demonstrated the neuroprotective effects from genetic knockdown or pharmacological inhibition of ASK1 after cerebral ischemia [21, 100, 101]. Neuroprotective drugs show beneficial effects by suppression of ASK1/JNK signals [102]. Based on previous studies, ASK1 may be involved in calcium influx, oxidative stress, neuronal cell death, and cerebral infarctions after ischemic stroke. (Table 1).

## 8. Conclusion

Ischemic stroke is a complex neurologic disorder with limited treatment options, which amplifies the need for drug development. This review focuses on cell type-specific pathomechanisms, mainly targeting platelets, endothelial cells, immune cells, astrocytes, and neurons in preclinical ischemic stroke models. We focus on ASK1 as a major target molecule in the etiology of ischemic stroke. Pharmacologic and genetic inhibition of ASK1 has been shown to provide neuroprotective effects in cerebral ischemia. Therefore, we would like to highlight the importance of ASK1 as a key target in drug development for ischemic stroke.

## Figures and Tables

**Table 1:** Genetic/pharmacological inhibition of ASK1. bEnd.3 cell line (mouse endothelial cells), BV2 cell line (mouse microglia cells), RAW 264.7 cell line (mouse macrophage cells), HAEC cells (human aortic endothelial cells), and MEF cells (mouse embryonic fibroblasts). MPO: myeloperoxidase; ICV: intracerebrovascular injection; IV: intravenous injection; IH: intrahemispheric injection.

Genetic/pharmacological inhibition of ASK1	Dosage	Application	Injury or duration	Main outcome	Reference
ASK1 knockout (KO)		ASK1 KO mice	Optic nerve injury	Eye: apoptotic cell death↓, MCP-1↓, TNF-*α*↓, iNOS↓	[77]
		MEF cells	H_2_O_2_ or TNF treatment	Apoptotic cell death↓	[96]
		Platelets		Thromboxane A2↓, platelet granule secretion↓	[22]
		ASK1 KO mice	FeCl_3_-induced injury	Carotid artery thrombosis↓	[22]
Small interfering RNA (siRNA) for ASK1	5 *μ*M/mice (ICV)	C57/BL6 mice	3 days before ischemic/reperfusion (I/R) injury	Brain: brain edema↓, VEGF↓, AQP-1↓	[24]
	5 *μ*M/mice (ICV)	C57/BL6 mice	3 days before I/R injury	Brain: neuronal cell death↓, MMP-9↓	[50]
	5 *μ*M/mice (ICV)	C57/BL6 mice	3 days before I/R injury	Brain: GFAP↓, glial scar formation↓	[19]
	5 *μ*M/mice (ICV)	C57/BL6 mice	3 days before I/R injury	Brain: IL-6↓,TNF-*α*↓, IL-1*β*↓	[23]
	5 *μ*M/mice (ICV)	C57/BL6 mice	3 days before I/R injury	Brain: infarct volume↓	[21]
Nanoparticles with anti-ASK1 short hairpin RNA (shRNA)	150 *μ*g/rat (IV)	Spraque-Dawley rats	2 days before I/R injury	Brain: apoptotic cell death↓, infarct injury↓	[101]
NQDI-1, ASK1 inhibitor	600 nM	bEnd.3 cell line	3 hrs before oxygen/glucose deprivation (OGD)	VEGF↓	[24]
	600 nM	bEnd.3 cell line	1 hr before OGD/6 hrs during OGD	Released MMP-9↓	[50]
	600 nM	BV2 cell line	1 hr before OGD/4 hrs during OGD	IL-6↓, TNF-*α*↓, IL-1*β*↓, iNOS↓	[23]
	600 nM	RAW 264.7 cell line	1 hr before OGD/4 hrs during OGD	IL-6↓, TNF-*α*↓, IL-1*β*↓, iNOS↓	[23]
	250 nmol/pup (IH)	Spraque-Dawley rats	30 mins before hypoxia-ischemia (HI) injury	Brain: caspase-3↓, infarct volume↓, apoptotic cell death↓	[100]
AGI-1067, ASK1 inhibitor	10 *μ*M	HAEC cells	1 hr before LPS/during LPS treatment	VCAM-1↓, E-selectin↓, IL-6↓, MCP-1↓	[73]
Thioredoxin	2 *μ*g/g (IV)	C57/BL6 mice	10 mins before ventilation	Infiltration of neutrophils↓, MPO↓	[75]
MSC2032964A, ASK1 inhibitor	10 *μ*M	Primary microglia cells	1 hr before LPS treatment	TNF-*α*↓, iNOS↓	[77]

## Abbreviations

CNS: Central nervous system
ASK1: Apoptosis signal-regulating kinase 1
CBF: Cerebral blood flow
ROS: Reactive oxygen species
MAPKKK: Mitogen-activated protein kinase kinase kinase
MAP2K: Mitogen-activated protein kinase kinase
MAPK: Mitogen-activated protein kinase
Trx: Thioredoxin
H_2_O_2_: Hydrogen peroxide
JNK: c-Jun N-terminal kinase
vWF: von Willebrand factor
ADP: Adenosine diphosphate
TXA_2_: Thromboxane A_2_
PLA_2_: Phospholipase A_2_
ERK2: Extracellular signal-regulated kinase 2
cPLA_2_: Cytoplasmic phospholipase A2
BBB: Blood-brain barrier
MMPs: Metalloproteinases
VEGF: Vascular endothelial growth factor
MCP-1: Monocyte chemoattractant protein-1
VCAM-1: Vascular adhesion molecule-1
ICAM-1: Intercellular adhesion molecule-1
NETs: Neutrophil extracellular traps
TLR: Toll-like receptor
IL-1*β*: Interleukin-1*β*
TNF-*α*: Transforming necrosis factor-*α*
TGF-*β*: Transforming growth factor-*β*
iNOS: Inducible nitric oxide synthase
LPS: Lipopolysaccharide
GFAP: Glial fibrillary acidic protein
BDNF: Brain-derived neurotrophic factor
NGF: Nerve growth factor
IFN*γ*: Interferon-gamma
NO: Nitric oxide
NMDA: N-Methyl-D-aspartate
AMPA: *α*-Amino3-hydroxy-5-methyl-4-isoxazolepropionic acid
CaMK II: Ca^2+^/calmodulin-dependent protein kinase II
nNOS: Nitric oxide synthase.
